# DIANA-microT 2023: including predicted targets of virally encoded miRNAs

**DOI:** 10.1093/nar/gkad283

**Published:** 2023-04-24

**Authors:** Spyros Tastsoglou, Athanasios Alexiou, Dimitra Karagkouni, Giorgos Skoufos, Elissavet Zacharopoulou, Artemis G Hatzigeorgiou

**Affiliations:** DIANA-Lab, Department of Computer Science and Biomedical Informatics, Univ. of Thessaly, Lamia 35131, Greece; Hellenic Pasteur Institute, Athens 11521, Greece; DIANA-Lab, Department of Computer Science and Biomedical Informatics, Univ. of Thessaly, Lamia 35131, Greece; Hellenic Pasteur Institute, Athens 11521, Greece; Department of Pathology, Beth Israel Deaconess Medical Center, Boston, MA, USA; Harvard Medical School, Boston, MA, USA; Broad Institute of MIT and Harvard, Cambridge, MA, USA; DIANA-Lab, Department of Computer Science and Biomedical Informatics, Univ. of Thessaly, Lamia 35131, Greece; Hellenic Pasteur Institute, Athens 11521, Greece; DIANA-Lab, Department of Computer Science and Biomedical Informatics, Univ. of Thessaly, Lamia 35131, Greece; Hellenic Pasteur Institute, Athens 11521, Greece; DIANA-Lab, Department of Computer Science and Biomedical Informatics, Univ. of Thessaly, Lamia 35131, Greece; Hellenic Pasteur Institute, Athens 11521, Greece

## Abstract

DIANA-microT-CDS is a state-of-the-art miRNA target prediction algorithm catering the scientific community since 2009. It is one of the first algorithms to predict miRNA binding sites in both the 3′ Untranslated Region (3′-UTR) and the coding sequence (CDS) of transcripts, with increased performance. Its current version, DIANA-microT 2023 (www.microrna.gr/microt_webserver/), brings forward a significantly updated set of interactions. DIANA-microT-CDS has been executed utilizing annotation information from Ensembl v102, miRBase 22.1 and, for the first time, MirGeneDB 2.1, yielding more than 83 million interactions in human, mouse, rat, chicken, fly and worm species. Additionally, this version delivers predicted interactions of miRNAs encoded from 20 viruses against host transcripts from human, mouse and chicken species. Numerous resources have been interconnected into DIANA-microT, including DIANA-TarBase, *plasmiR*, HMDD, UCSC, dbSNP, ClinVar, as well as miRNA/gene abundance values for 369 distinct cell-lines/tissues. The server interface has been redesigned allowing users to use smart filtering options, identify abundance patterns of interest, pinpoint known SNPs residing on binding sites and obtain miRNA-disease information. The contents of DIANA-microT webserver are freely accessible and can also be locally downloaded without any login requirements.

## Graphical Abstract

**Graphical Abstract:**
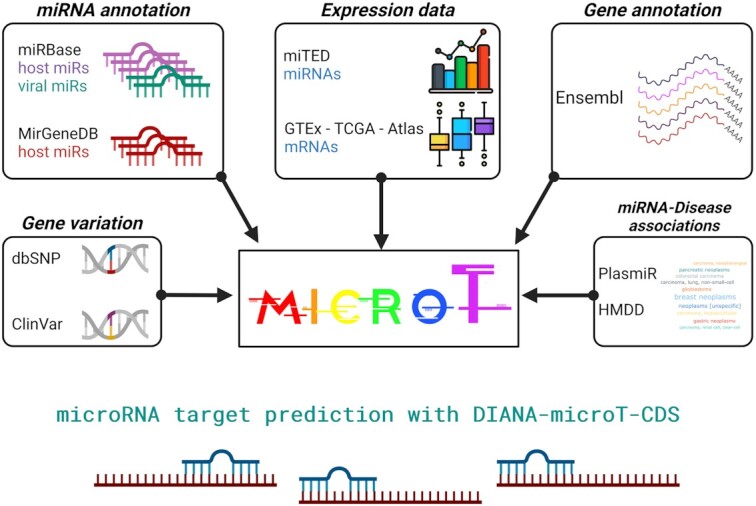
DIANA-microT 2023 couples miRNA target predictions from various annotation sources with expression-, variant- and disease-relevant data in a redesigned webserver. Created with BioRender.com.

## INTRODUCTION

microRNAs (miRNAs) are short non-coding RNAs that post-transcriptionally target miRNA recognition elements (MREs) in the 3′ untranslated region (3′-UTR) (1) and the coding sequence (CDS) (2) of transcripts, primarily inducing transcript degradation and/or protein synthesis stall. The many-to-many relationship that miRNAs exhibit with messengers and other RNA species (e.g. long non-coding RNAs (3), circular RNAs (4)) place them centrally to the post-transcriptional regulatory nexus and deem them important players in finetuning potentially all biological processes.

Besides miRNA genes residing in host species’ genomes, notably, miRNAs encoded by viruses (v-miRNAs) have been identified and are supported by experimental evidence (5–8). The miRBase Registry currently holds sequence information about approximately 530 mature v-miRNAs encoded in genomes of 34 viruses (predominantly herpesviruses) (9). v-miRNA biogenesis occurs using the host's miRNA machinery (10). Viral targetome studies, focused on Epstein-Barr virus (EBV) and Kaposi sarcoma-associated herpesvirus (KSHV), indicate the implication of v-miRNAs in regulating the host transcriptome aiding infection, maintaining viral latency and inducing pathogenesis (11,12).

Despite the relative wealth in available experimentally verified interactions (13,14), miRNA target prediction remains relevant; for a number of species, states and annotation schemes (e.g. the reference miRNA database MirGeneDB (15)) there is limited to no experimental support for miRNA targets. In such scenarios, target prediction may be the only way to guide downstream experimental and computational investigations of miRNAs’ function and roles.

The rules that dictate effective (host and viral) miRNA binding are still being extensively studied. Accumulating evidence produced during the past two decades delineates potent features that can be utilized to detect robust and efficacious MREs (16–20). These include characteristics regarding (a) the site accessibility on the candidate MRE, (b) thermodynamic properties of the MRE and its flanking sequence, (c) the MRE position within the 3′-UTR/CDS, (d) the sequence composition of the MRE, as well as of its flanking regions, (e) the miRNA-MRE duplex stability, described in thermodynamics terms, biochemical stability terms, as well as through the number of matches, mismatches, wobble pairs and bulges that characterize the miRNA seed binding (positions 2–7 from the miRNA’s 5′-end) and the entire binding, as well as (f) conservation metrics of the binding sites.

DIANA-microT-CDS (21) was one of the first target prediction algorithms to integrate PAR-CLIP (photoactivatable ribonucleoside-enhanced crosslinking and immunoprecipitation followed by high-throughput sequencing)-derived data during its training and testing, and to be composed of two distinct models trained separately for the 3′-UTR and the CDS sequences. The previous version of the DIANA-microT webserver (22) focused on updating the resources used to perform predictions and providing a number of services that are currently covered by other DIANA-Tools online applications (23,24).

In this version we deliver an extensive set of microT-CDS-predicted interactions based on updated annotations of miRNAs and genes. DIANA-microT 2023 provides interactions utilizing miRBase (9) and MirGeneDB (15) as sources for miRNA sequences for human, mouse, rat, chicken, fly and worm species. Additionally, a specialized set of v-miRNA interaction predictions against host genes is also provided for viruses that infect human (14 viruses, 196 v-miRNAs), mouse (2 viruses, 57 miRNAs) and chicken (4 viruses, 100 miRNAs) which have been annotated by miRBase as miRNA-encoding. The positions of known SNPs from the reference resources dbSNP (25) and ClinVar (26) have been overlapped with predicted MREs, to indicate instances where predicted miRNA targeting efficacy could be altered by variants. We integrated miRNA abundance estimates regarding 60 tissues and 210 cell-lines derived from the miRNA Tissue Expression Database, DIANA-miTED (27) and gene expression for 99 human and mouse contexts from the Genotype-Tissue Expression project (GTEx) (28), The Cancer Genome Atlas (TCGA) (29) and the reference mouse publication by Sollner et al. (30), to make possible the incorporation of abundance information in returned interactions. Finally, human miRNAs are annotated regarding their causal associations with diseases from HMDD resource (31), as well as with their capacity to function as circulating miRNA biomarkers, from *plasmiR* (32). The DIANA-microT 2023 webserver interface has been upgraded, and useful filtering and query functionalities have been set up to facilitate browsing through the enhanced content.

## METHODS AND RESULTS

### DIANA-microT-CDS executions

Ensembl v102 (33) was used as a reference for transcript annotations, selecting for prediction protein-coding genes on the standard chromosomes, while miRNA sequences and annotations were obtained through miRBase v22.1 (9) and MirGeneDB 2.1 (15) resources. Multiple sequence alignments were obtained through UCSC genome browser (34) for the corresponding genome assemblies (i.e. hg38, 30-way for *H. sapiens*, mm10, 60-way for *M. musculus*, rn6, 30-way for *R. norvegicus*, galGal6, 77-way for *G. gallus*, dm6, 27-way for *D. melanogaster* and WBcel235/ce11, 26-way for *C. elegans*). Secondary structure predictions were calculated for CDS and UTR regions using Sfold (35). microT-CDS was executed for each species for miRBase and MirGeneDB annotations, yielding in total approximately 83.9 million interactions (5.9 million interactions at the default microT score threshold of 0.7). Additionally, predictions of v-miRNA sequences were performed for human (viruses: Epstein–Barr virus (EBV), Kaposi's sarcoma-associated herpesvirus (KSHV), Human cytomegalovirus (HCMV), Herpes Simplex Virus 1 (HSV1), Herpes Simplex Virus 2 (HSV2), Human Immunodeficiency Virus (HIV1), Human Herpesvirus 6B (HHV6B), Merkel cell polyomavirus (MCPV), Human polyomavirus 2 (JCV), BK polyomavirus (BKV), Torque teno virus (TTV), Simian foamy virus (SFV), Herpes B virus (HBV) and Simian virus 40 (SV40)), mouse (viruses: Murine gammaherpesvirus 68 (MGHV) and Mouse cytomegalovirus (MCMV)), and chicken (viruses: Marek's disease virus serotype 2 (MDV2), Marek's disease virus serotype 1 (MDV1), Turkey herpesvirus (HVT) and Infectious laryngotracheitis virus (ILTV)). The provided v-miRNA entries correspond to ∼2.5 million interactions (185 460 with microT score at least 0.7). Brief metrics of the updated DIANA-microT 2023 webserver content are provided in Table 1.

**Table 1.** Total number of miRNAs, predicted interactions and corresponding MREs that are catered through DIANA-microT 2023 webserver (miRBase and MirGeneDB denoted as ‘MB’ and ‘MG’ respectively). The first section refers to species’ miRNA–gene interactions, while the second provides metrics regarding v-miRNAs of each included virus

		Interactions		MREs	
Species	miRNAs (MB/MG)	MB	MG	MB	MG
**Human**	2656/1133	25.2 × 10^6^	10.5 × 10^6^	58 × 10^6^	23.5 × 10^6^
**Mouse**	1978/903	17.9 × 10^6^	7.9 × 10^6^	36.1 × 10^6^	15.5 × 10^6^
**Rat**	764/838	4.2 × 10^6^	4.6 × 10^6^	6.7 × 10^6^	7.3 × 10^6^
**Chicken**	1235/570	6.3 × 10^6^	2.8 × 10^6^	10.2 × 10^6^	4.4 × 10^6^
**Fly**	469/322	1.6 × 10^6^	10^6^	2.5 × 10^6^	1.6 × 10^6^
**Worm**	437/289	1.2 × 10^6^	0.8 × 10^6^	1.5 × 10^6^	10^6^
**Total**	7539/4055	56.3 × 10^6^	27.7 × 10^6^	114.9 × 10^6^	53.2 × 10^6^
**EBV (human)**	44/–	383.1 × 10^3^	–	859.3 × 10^3^	–
**SFV (human)**	13/–	138 × 10^3^	–	433 × 10^3^	–
**KSHV (human)**	25/–	219 × 10^3^	–	479.2 × 10^3^	–
**HCMV (human)**	26/–	189.2 × 10^3^	–	388.7 × 10^3^	–
**HSV1 (human)**	27/–	228.8 × 10^3^	–	494.7 × 10^3^	–
**HBV (human)**	15/–	135.5 × 10^3^	–	313.9 × 10^3^	–
**HSV2 (human)**	24/–	166.5 × 10^3^	–	323.7 × 10^3^	–
**HIV1 (human)**	4/–	45.3 × 10^3^	–	115.8 × 10^3^	–
**HHV6B (human)**	8/–	44.6 × 10^3^	–	86.4 × 10^3^	–
**MCPV (human)**	2/–	25.1 × 10^3^	–	61.6 × 10^3^	–
**JCV (human)**	2/–	22.4 × 10^3^	–	50.3 × 10^3^	–
**BKV (human)**	2/–	21 × 10^3^	–	43.4 × 10^3^	–
**SV40 (human)**	2/–	20.8 × 10^3^	–	47.5 × 10^3^	–
**TTV (human)**	2/–	16.7 × 10^3^	–	31.4 × 10^3^	–
**MGHV (mouse)**	28/–	230.5 × 10^3^	–	436.8 × 10^3^	–
**MCMV (mouse)**	29/–	193.9 × 10^3^	–	362.2 × 10^3^	–
**MDV2 (chicken)**	36/–	177.4 × 10^3^	–	288 × 10^3^	–
**MDV1 (chicken)**	26/–	114.4 × 10^3^	–	188.2 × 10^3^	–
**HVT (chicken)**	28/–	128.3 × 10^3^	–	217.5 × 10^3^	–
**ILTV (chicken)**	10/–	52.8 × 10^3^	–	84.3 × 10^3^	–
**Total (viral)**	353/–	2.6 × 10^6^	–	5.3 × 10^6^	–

### Supplementary data collection

  **Conservation**. Corresponding phastCons BigWig files were derived from UCSC for each species. Mean phastCons per MRE were calculated using *bigWigAverageOverBed* from UCSC Utilities (34,36,37).

**Additional interaction support**. Experimentally verified interactions from DIANA-TarBase v8.0 (13) and TargetScan (19) predictions were obtained and matched to DIANA-microT-CDS predictions.

**Abundance information**. Gene expression data for human and mouse were obtained from GTEx (28), TCGA (29) and the Sollner *et al.* mouse expression atlas (30), resulting in 99 distinct tissue states. Raw read counts were transformed to transcripts-per-million. The median TPM across replicates was estimated for each state and log_2_-transformed after adding one. *z*-scores, denoting the distance in standard deviations of the gene's expression from the mean of expressions within each state, were calculated and annotated to genes. Similarly, summarized miRNA abundance estimates were obtained through DIANA-miTED (27) as Reads-Per-Million (RPM) values. They correspond to 270 cell-line/tissue states in human (miRBase annotation only; non-viral miRNAs). Log_2_-transformation and *z*-score scaling was again applied for each state.

**Variant annotations**. SNP VCF files were retrieved from dbSNP v151 (25) and ClinVar (26) (last accessed on November 13, 2022). SNPs were overlapped with MREs using *GenomicRanges*, resulting in 6355001 overlaps of 968339 SNPs with 3970444 MREs in total.

**Disease information**. Causal associations of miRNAs against diseases were retrieved from HMDD 3.2 (31). Circulating miRNA biomarker information was derived from *plasmiR* (32). For each miRNA, the number of HMDD or *plasmiR* entries supporting the association or biomarker capacity respectively was tallied and utilized to create miRNA disease clouds, implementing active hyperlinks towards these external resources.

### Implementation

The ***microT-webserver*** utilizes the Model-View-Controller (MVC) software architecture as its basis through use of the Laravel 8 PHP framework, and a RESTful interface to communicate with the Angular-based frontend. The webserver is hosted on an Apache 2.4 HTTP server while data are stored in a relational database managed by a PostgreSQL 11.8 server. The PHP framework Laravel 8 (https://laravel.com/) (PHP 7.2) handles the back-end logic including the connection to the PostgreSQL server for the storing and retrieval of the data. The front-end is designed as a one-page website using Angular 14 (https://angular.io/), employing the Angular Material UI library (https://material.angular.io/) and the ngx-bootstrap (https://valor-software.com/ngx-bootstrap) framework for its visual and functional components. Finally, database statistics are presented using the Chart JS (https://www.chartjs.org/) library, while AnyChart (https://www.anychart.com/) is utilized for the word-cloud visualizations provided to portray the frequency of diseases associated with specific miRNAs and provide active hyperlinks towards HMDD and *plasmiR* servers.

### Interface and functionality

**Figure 1.**
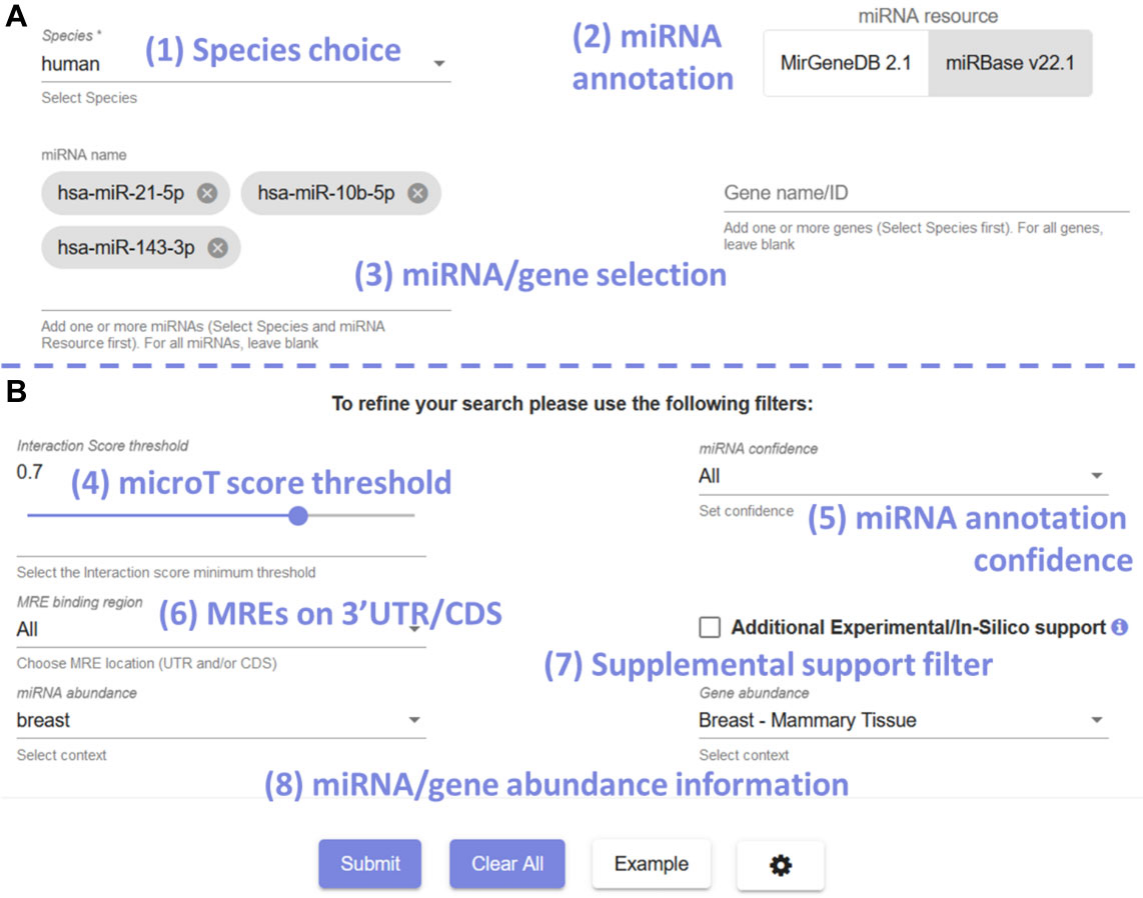
DIANA-microT 2023 input interface. (**A**) The primary query interface for interactions. After selecting (**1**) the species and (**2**) the miRNA annotation of interest, users can instantly perform queries by providing (**3**) on one/multiple mature miRNA names and/or Gene Symbols or Ensembl Gene IDs. (**B**) If required, they may expand the query device to reveal further filtering and annotation options. (**4**) The microT score threshold can be manually set, while (**5**) the option to limit output to miRNA entries annotated as ‘Highly confident’ by miRBase (MirGeneDB miRNAs are all annotated as ‘Highly confident’) can be employed. Users may also (**6**) retain only interactions and MREs predicted on the 3′UTR or the CDS of transcripts, (**7**) require that output has supplemental experimental (DIANA-TarBase) or predicted (TargetScan) support, and (**8**) select among available tissues and cell-lines to derive additional information regarding the abundance of miRNAs and/or genes specifically there.

The previous DIANA-microT version featured a minimal interface to assess miRNA target predictions, consisting of a miRNA/gene input service and a filter to manually set the prediction score threshold. This philosophy is inherited in the interface of DIANA-microT 2023 webserver, enabling fast and easy retrieval of predictions of interest (Figure 1A). The basic menu requires a target species to be set, a miRNA annotation source to be selected and any number of miRNAs to be provided. Yet, if required, users may unfold a supplemental menu that allows more sophisticated queries to be performed (Figure 1B). Apart from the minimum prediction score, the capacity to derive interactions only supported by highly confident miRNA annotations, output only MREs on the CDS or 3′-UTR, or output only interactions that are also supported by TarBase and/or TargetScan is offered. Importantly, two dedicated controls enable selection among the available tissues and cell-lines, annotating the results with abundance information on them.

**Figure 2.**
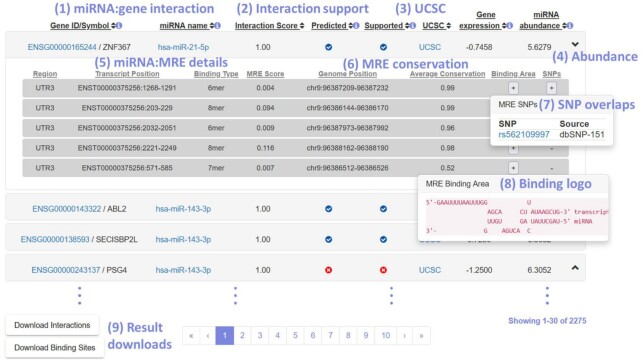
DIANA-microT 2023 output format. The provided output is organized into a paginated-expandable list of results. The first layer of information includes (**1**) the interacting miRNA and gene, which can be selected to reveal/hide supplemental details. For genes, these include the Gene Description, the representative transcript ID, the Ensembl version and hyperlinks towards TarBase and Ensembl. For miRNAs, the miRNA sequence, links towards miRBase/MirGeneDB, plasmiR, DIANA-miTED, and informative word-clouds based on causal disease associations (HMDD) and miRNA biomarkers (plasmiR) are offered. (**2**) Information regarding interaction score and supplemental support from other sources, (**3**) a hyperlink towards the UCSC Genome Browser and, if available, (**4**) abundance metrics in a specific context are available. Expanding the view, MRE-level details, including (**5**) the transcript region, site coordinates, binding type, (**6**) average conservation of the MRE, (**7**) its overlap with known SNPs, as well as (**8**) a text-based depiction of each binding area can be viewed. (**9**) Interaction- or MRE-level results may be retrieved locally in tab-delimited format, while the entire set of interactions per-species is available for download in a separate dedicated tab.

The generated interactions table has been refurbished to provide all associated information in an intuitive hierarchical schema (Figure 2). The primary layer provides interaction-level details; each miRNA–gene pair is accompanied by its interaction score, notation to indicate further predicted/experimental support, a link pointing to a dedicated UCSC track with all interaction-specific MRE positions and *z*-scores of abundance metrics in case specific expression contexts have been selected for genes and/or miRNAs. Abundance metrics can be used to provide context-specific experimental support, e.g. hsa-miR-143-3p in the example is highly expressed relative to other miRNAs (6.3 standard deviations higher than the miRNAs’ mean) and PSG4 appears to be moderately repressed (1.25 standard deviations lower than the genes’ mean). Gene identifiers and miRNA names can be clicked to reveal gene- and miRNA-level details, including causal association and biomarker disease-clouds for each miRNA. Supplemental information on the MRE level is provided by expanding an entry of interest. The region (3′-UTR or CDS), MRE coordinates on the respective transcript/genome, primary binding type and MRE score are the basic MRE details. Additionally, the average phastCons conservation of the predicted MRE is provided and pop-up buttons enable retrieving a schematic of the MRE binding area and information about SNPs overlapping the MRE sequence.

## CONCLUSION

As miRNA research progresses, novel miRNA annotation efforts are made available and gradually become accepted by the community. Similarly, even though viral miRNAs have been discovered and annotated in the past, their targetomes remain elusive and have been mostly studied experimentally for a limited set of prominent viruses, such as EBV and KSHV, and v-miRNAs. These valuable miRNA sets can have limited utility if *in silico* and wet-lab approaches fall short of integrating them. miRNA target prediction constitutes a missing link towards the effective assessment of their interactomes, their further functional investigation and their proper incorporation into downstream experimental studies. Importantly, miRBase and MirGeneDB can exhibit sequence differences (both on the 5′- and the 3′-end) even in mature miRNAs that they both provide, with immediate effects on the corresponding targeting repertoires. DIANA-microT 2023 webserver bridges this gap by delivering a service of miRNA interactions for both miRBase and MirGeneDB, as well as predictions of v-miRNA interactions with host transcripts. This major upgrade is enveloped into a newly designed interface offering new functionalities, interconnections with other tools and unrestricted retrieval capacity.

## DATA AVAILABILITY

DIANA-microT 2023 server is accessible freely and without login requirements (www.microrna.gr/microt_webserver, www.microrna.gr/webServer). Query results, as well as the entire set of interaction predictions, are also available for local retrieval through the application.
